# Experimental Data on design, theoretical and correlation of the electronic and optical properties of diethynylphenylthiophene as photovoltaic materials

**DOI:** 10.1016/j.dib.2020.105579

**Published:** 2020-04-23

**Authors:** Yenny Ávilla Torres, Melissa Suarez, Carolina Caicedo, Hoover Valencia, Edwin Flórez-López

**Affiliations:** 1 Grupo QUIBIO, Facultad de Ciencias Básicas, Universidad Santiago de Cali, Santiago de Cali, Colombia; 2 Grupo Fotocatálisis y estado sólido, QCOAMMSB, Universidad Tecnológica de Pereira, Pereira, Colombia

**Keywords:** Photosensitized materials, Acceptor- donor structure, Solar applications

## Abstract

The article show the date associated with the work previously reported “Design, theoretical and correlation of the electronic and optical properties of diethynylphenylthiophene as photovoltaic materials”, https://doi.org/10.1016/j.molstruc.2019.127093[1]. The authors reported graphics and tables building from of p-PDT, m-PDT, o-PDT, p-ZnPDT, m -ZnPDT and o-ZnPDT calculations as raw date, with the aim of to show electronic and optical properties, which can be analyzed by the reader. In this context, there exists an important number of renewable energies that are substituting the oil and the charcoal be used in the energetic supply. One of these alternatives is the use of solar cells, which can be use in diverse areas like telecommunications, remote systems of monitoring, lighting systems, water treatment systems, and products of consumption. The employment of the organic photovoltaic technology and photosensitized organic materials are based on the use of molecular organic materials for coverings for ceiling and windows of a house that allow the storage of energy. The OPVs and DSSC present π conjugated systems, giving them a high electronic relocated density, which allows catching the radiations with an energy range of wavelengths between 400 and 800 nm. The systems are derived of diethynylphenylthiophene (LMWOM) coupled to phenyldiamine (PD) as spacer, forming hyper conjugated macrocycles (*p*-PDT, *m*-PDT, *o*-PDT, *p*-ZnPDT, *m* -ZnPDT and *o*-ZnPDT). On the other hand, it is reported process electronic relationship with material sensitized and the bibliographic support of the publication topic.

Specifications table

**Table:** 

Subject	Organic Chemistry
Specific subject area	Science Materials
Type of data	Tables and Figures
How data were acquired	Spectroscopic characterization (UV- Vis) DFT data (Bond distances and angles of optimized molecules) Structural strategies in photosensitized materials with potential applications in solar cells.
Data format	Raw
Parameters for data collection	The information is obtained from the raw data derived from Gaussian 09 computing program, which can be analyzed by the reader. The authors reported bond lengths for *p*-PDT, *m*-PDT and *o*-PDT and *o*-ZnPDT and *m*-ZnPDT. This allows to interpret the specific effect for atom by presence of zinc(II). Also, the directionally of the dipole moment is shown, the donor - acceptor map in relationship to attacks environmental. The dipole moment allows establishing the planarity of the molecules, and comparing them with similar ones.
Description of data collection	Angles and dipolar moment associated to lineal Molecule (LMWOM (1), moment dipolar associated to macrocycles with different spacers a). *o*-PDT, b). *m*-PDT and c). *p*-PDT, optimization of lineal molecule coordinated Lewis acid (angles, structure molecular, HOMO- LUMO description) and Donor- Acceptor capacity for macrocycles studied in relationship with Reactive Species capacity, which can degrade in outdoors conditions, also reported. The readers can calculate the GAP according to the acceptor capacity and if they wish to apply these materials in the photovoltaic cell industry, they can estimate their corrosion or damage by agents such as hydroxyl radicals. Also, the authors show a revision of molecules associated with applications in solar cells, reporting data which the reader can compare the optical and electronic properties, with final results in https://doi.org/10.1016/j.molstruc.2019.127093. Finally, a new synthesized molecule is proposed, for which the data have not been analyzed and is a striking molecule for readers.
Data source location	Institution: Universidad Santiago de Cali
Data accessibility	The data are found only in this article M. Suarez, C. Caicedo, J. Morales, E. Florez- López, Y. Ávila- Torres, Design, theoretical study and correlation of the electronic and optical properties of diethynylphenylthiophene as photovoltaic materials. Journal of Molecular Structure*,* 2020, 127093 [1].
Related research article	M. Suarez, C. Caicedo, J. Morales, Flórez- López E, Ávila- Torres Y. Design, theoretical study and correlation of the electronic and optical properties of diethynylphenythiophene as photovoltaic materials, Journal of Molecular Structure 2020, 127093. https://doi.org/10.1016/j.molstruc.2019.127093

## Value of the Data

• These data are important because the distances and complete angles are reported, which have not been treated in relation to a new molecule derived from diethynylphenylthiophene. Likewise, the authors proposed other molecule derived with benzothiphene (BT), which could have best photovoltaic properties.
• The authors reported theoretical data for precursor molecules of macrocycles, the reader can stablish isomeric effects on the photovoltaic properties and improve the design of new molecules in the field.
• The readers can perform new theoretical calculations matching the macrocycles from diethynylphenyltiophene and benzothiphene (BT) considering o- m and p- phenyldiamine as spacer.
• These molecules can be used as new biomimetic materials to biological macrocycles as porphyrin. This macrocycle allows electronic transport using the metallic ion: iron. The readers can compare the electronic properties with other transition metal in configuration d10, such as: zinc(II).

## 1 Data

**Fig. 1:**
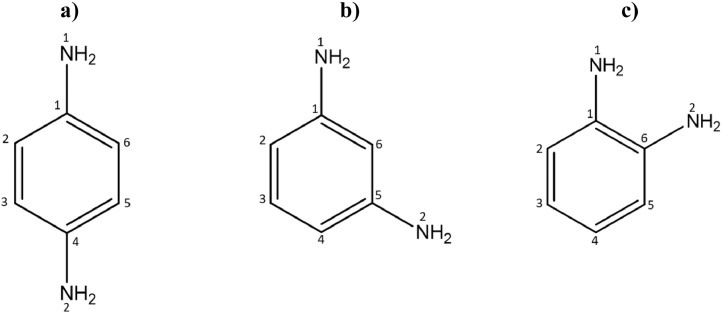
Chemical structure for a). *p*-Phenylenediamine (p- PD), b). *m*-Phenylenediamine (m- PD) and c). *o*-Phenylenediamine (o- PD)

**Fig. 2:**
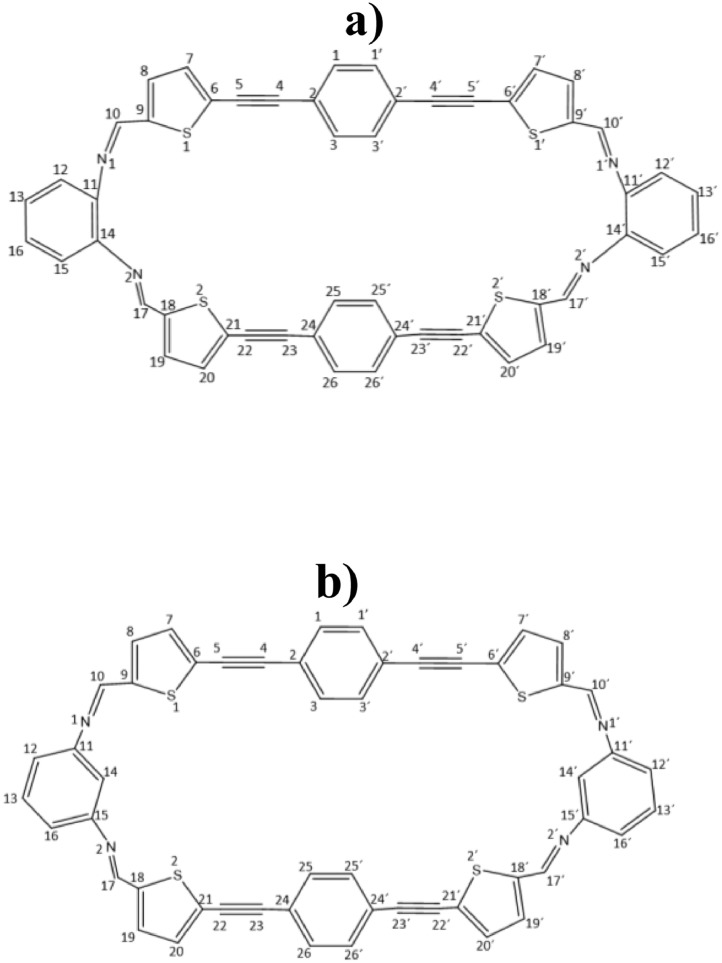
Chemical structure for a) *o*-PDT and b). *m*-PDT

**Fig. 3:**
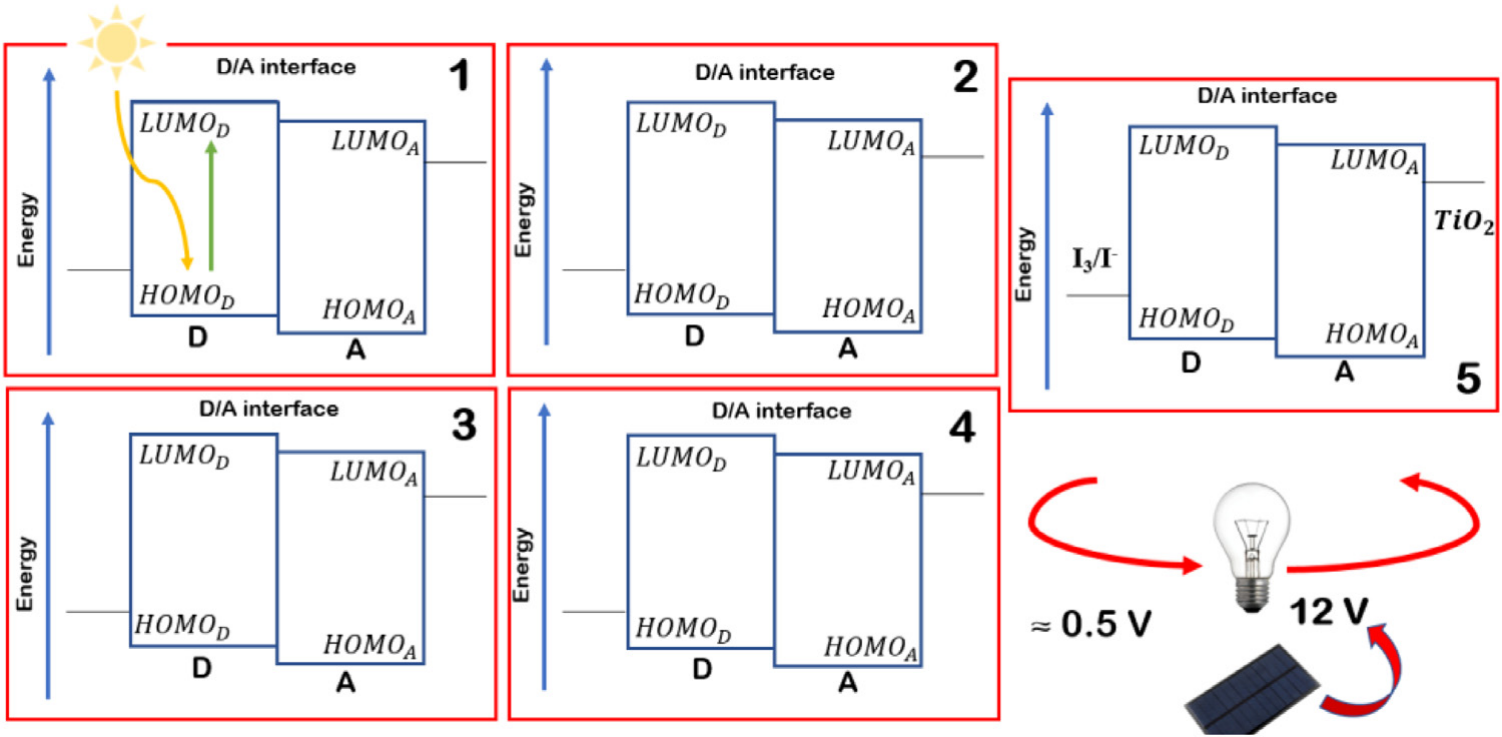
Scheme of electronic traffic through a sensitized material

**Fig. 4:**
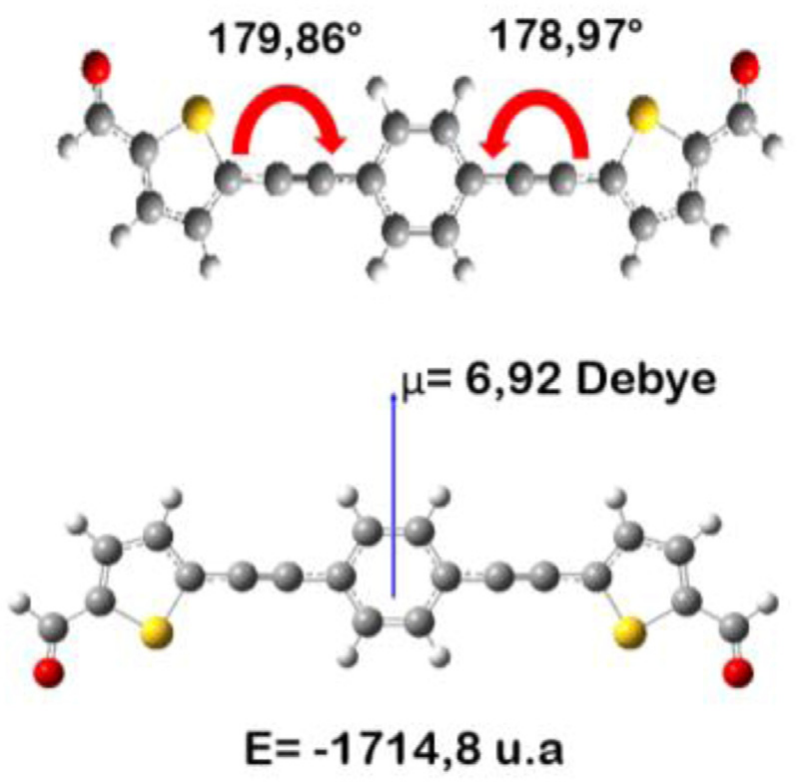
Angles and dipolar moment associated to lineal Molecule (LMWOM (1))

**Fig. 5:**
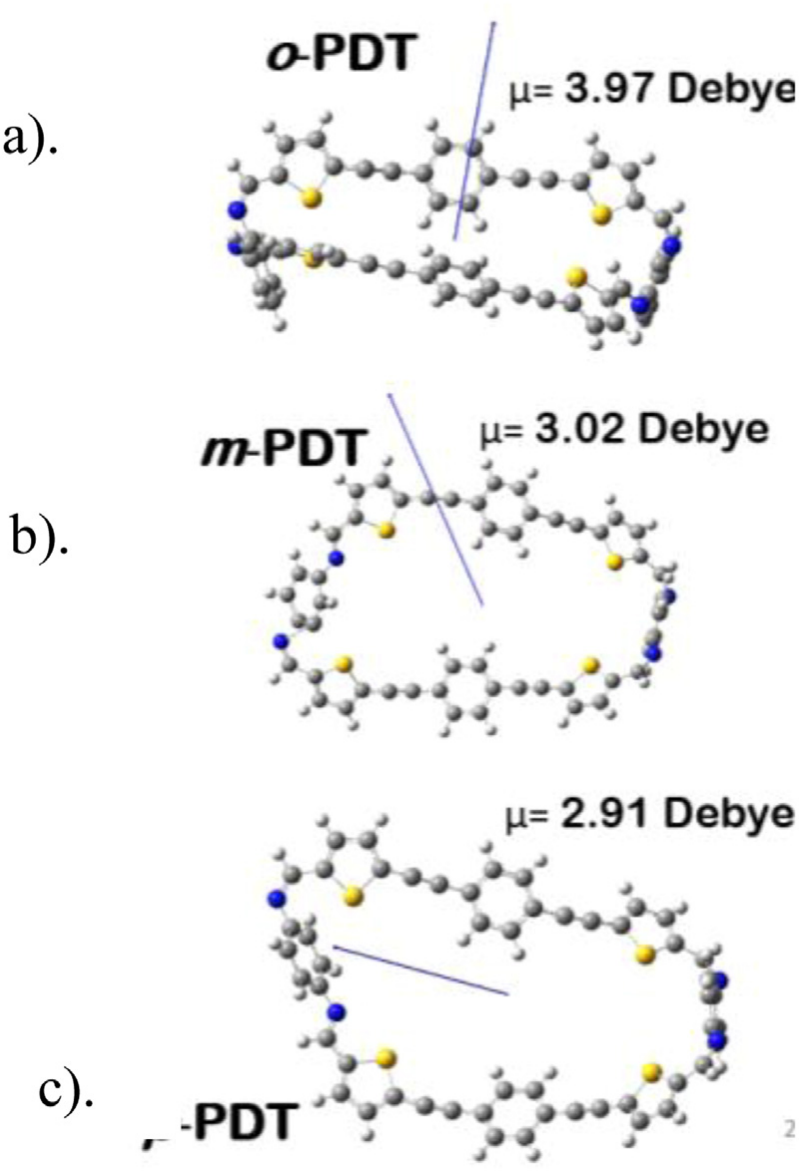
Moment dipolar associated to macrocycles with different spacers a). *o*-PDT, b). *m*-PDT and c). *p*-PDT.

**Fig. 6:**
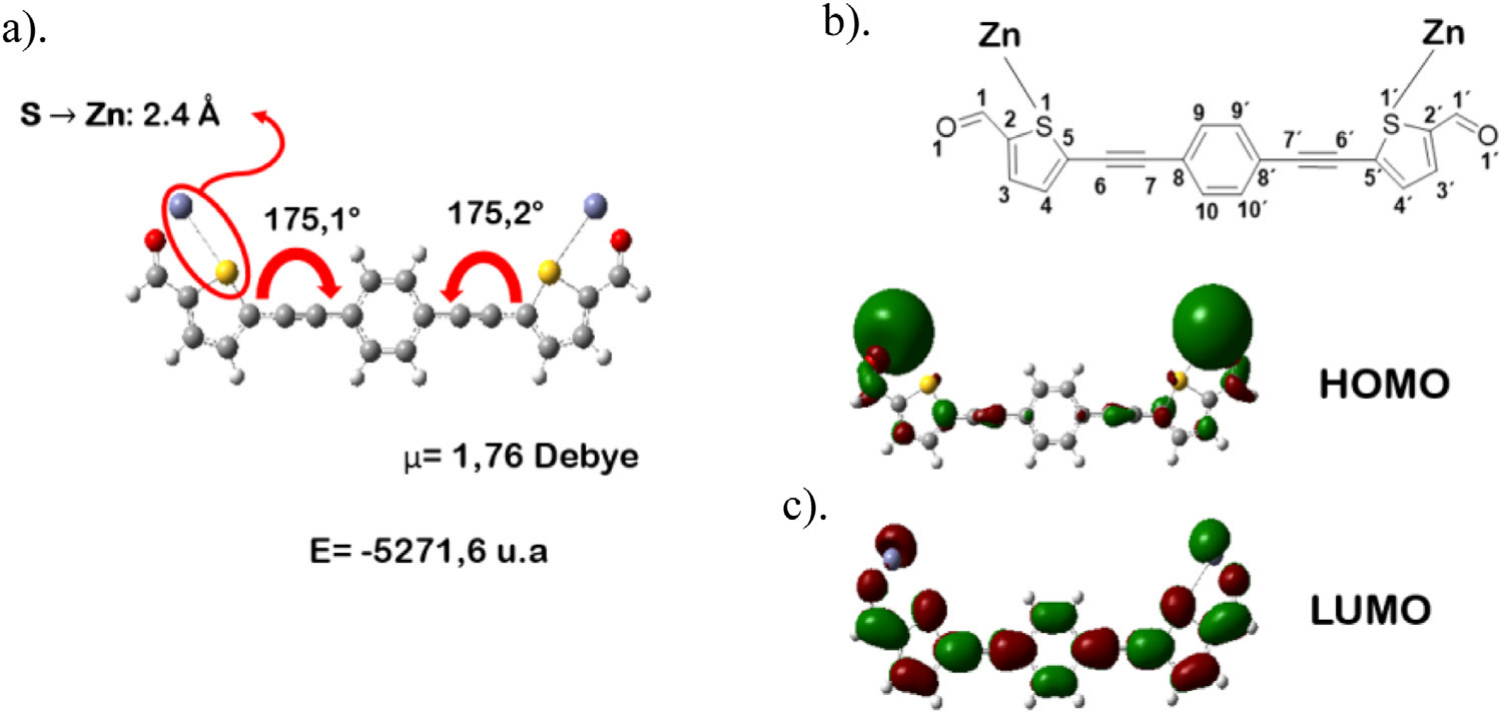
Optimization of lineal molecule coordinated Lewis acid, a). Angles, b). Structure molecular, c). HOMO- LUMO description

**Fig. 7:**
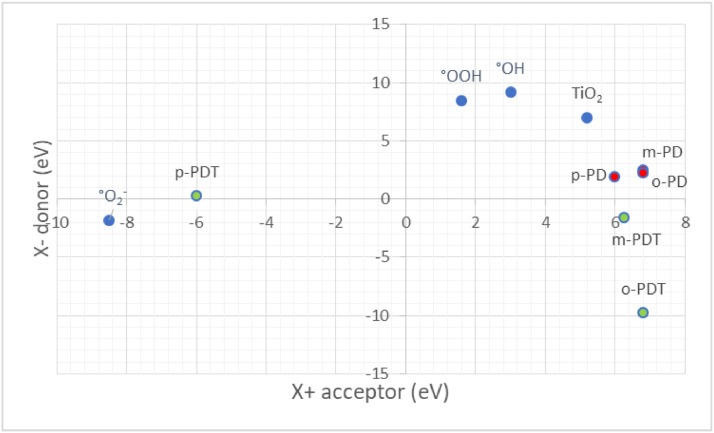
Donor- Acceptor capacity for macrocycles studied in relationship with Oxygen Reactive Species capacity, which these compounds in can degrade in outdoors conditions.

**Fig. 8:**
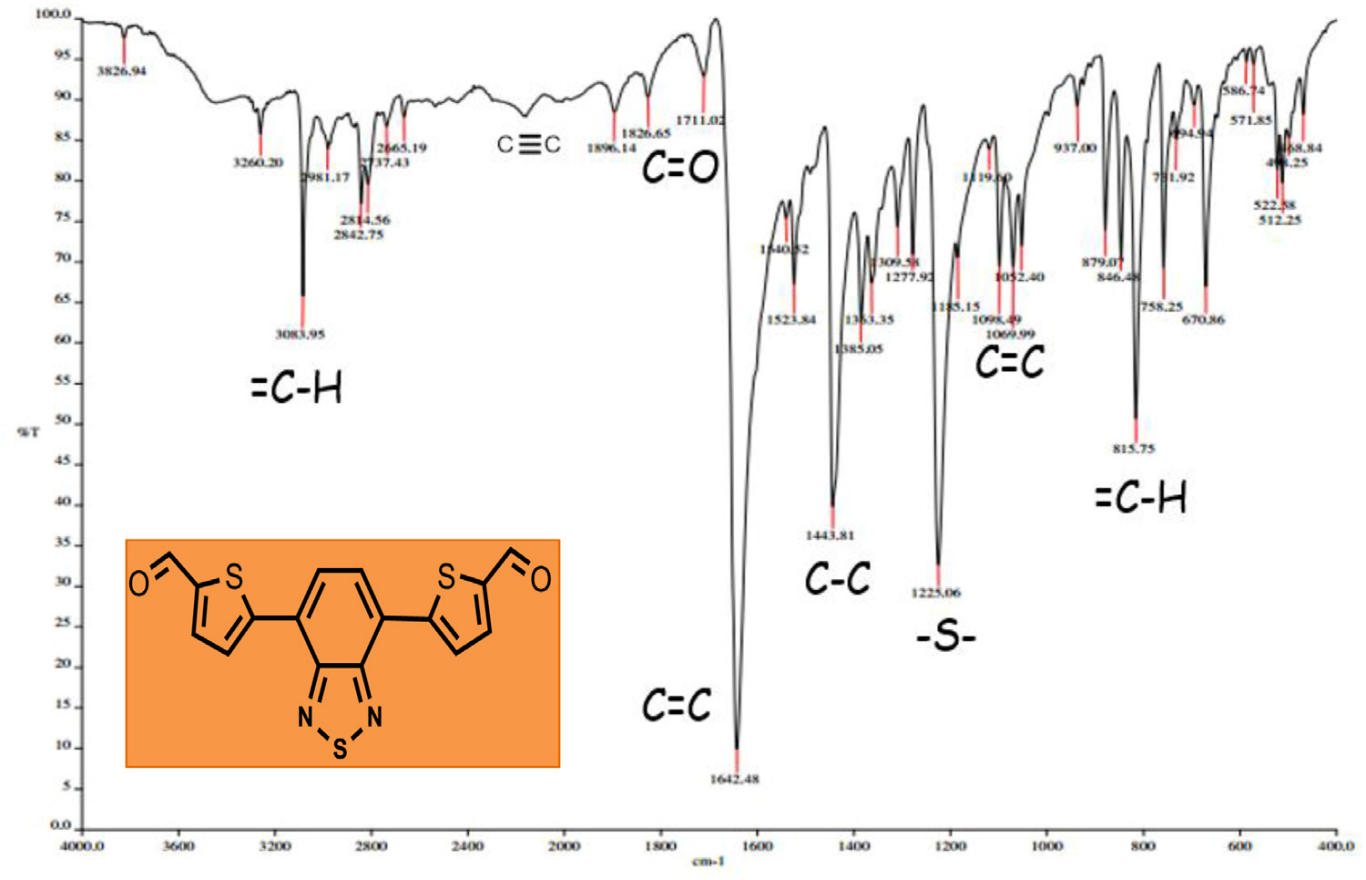
IR spectrum of Synthetized molecule as potential photovoltaic materials derivate of diethynylphenylthiophene (BT)

**Fig. 9:**
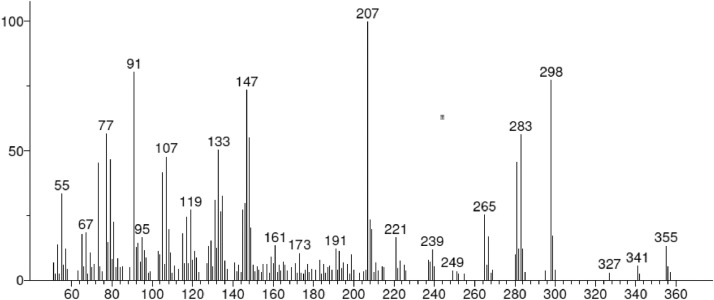
Mass spectrum m/z for the new molecule derivated of diethynylphenylthiophene (BT)

The distances and angles associated to the structure were calculated with the minimum energy in each case, for each optimized spacer and its respective macrocycle, Fig. 1 and 2, Table 1 and 2. The effect of Lewis acid is observed in the Table 3, in where were reported angles and distances associated to molecule optimized with these conditions. In the Fig. 3 is described the electronic process in a sensitized material, by means of which electronic transport occurs in this type of molecules. In the Fig. 4, the vector relationship with dipolar moment is showed for LMWOM (1) and macrocycles, which facilities la visibility on a plane specific, Fig. 5 and 6. The Lewis effect for lineal molecule is observed in the Fig. 7, stabilizing angles, structure molecular and HOMO- LUMO orbitals and its donor and acceptor capacity in sensitized molecules under typical environmental conditions. In the Table 4, the authors show the graphical comparison between molecule reported previously and new molecule synthesized in relation to electronic excitations, with the aim the readers can analyse of date and establish structural correlations. Likewise, in the Fig. 8, the IR spectrum of Synthetized molecule as potential photovoltaic materials derivate of diethynylphenylthiophene and Fig. 9, the mass spectrum m/z for the new molecule derivated of diethynylphenylthiophene, which has been proposed. Finally, in the Table 5 and 6 is reported the evolution in photosensitised materials with similar structural to the molecules synthesized, and the readers realize structural comparisons for to obtain best photovoltaic parameters.

**Table 1:** Bond lengths for *p*- PD, *m*- PD and *o*- PD.

BOND	p-PD	BOND	m-PD	BOND	o-PD
N1-C1	1.40995	N1-C1	1.40098	N1-C1	1.41077
C1-C2	1.40212	C1-C2	1.40516	C1-C2	1.39621
C2-C3	1.39240	C2-C3	1.39239	C2-C3	1.39703
C3-C4	1.40212	C3-C4	1.39246	C3-C4	1.39289
C4-N2	1.40997	C4-C5	1.40510	C4-C5	1.39708
C4-C5	1.40211	C5-N2	1.40084	C5-C6	1.39615
C5-C6	1.39242	C5-C6	1.40151	C6-N2	1.41086
C6-C1	1.40210	C6-C1	1.40141	C6-C1	1.41443
**ANGLE**	**p-PD**	**ANGLE**	**m-PD**	**ANGLE**	**o-PD**
N1-C1-C6	121.09033	N1-C1-C6	120.46212	N1-C1-C6	117.73855
C2-C1-N1	121.08921	C2-C1-N1	120.18014	C2-C1-N1	117.74929
C5-C4-N2	121.08974	C5-C6-N2	120.17260	C5-C6-N2	122.91470
C3-C4-N2	121.08967	C5-C4-N2	120.47366	C1-C6-N2	122.90703

**Table 2:** Bond lengths for **o-PDT** and **m-PDT**

BOND	o-PDT	BOND	o-PDT
C1-C2	1.41050	C1′-C2′	1.40955
C2-C3	1.41198	C2′-C3′	1.41360
C2-C4	1.42053	C2′-C4′	1.42036
C4-C5	1.21882	C4′-C5′	1.21970
C5-C6	1.40409	C5′-C6′	1.40432
C6-C7	1.38497	C6′-C7′	1.38814
C6-S1	1.75934	C6′-S1′	1.75211
C7-C8	1.41023	C7′-C8′	1.41382
C8-C9	1.38572	C8′-C9′	1.38233
S1-C9	1.74889	S1′-C9′	1.74700
C9-C10	1.46061	C9′-C10′	1.45101
C10-N1	1.27931	C10′-N1′	1.28328
N1-C11	1.39735	N1′-C11′	1.40788
C11-C12	1.40467	C11′-C12′	1.40203
C12-C13	1.39364	C12′-C13′	1.39185
C13-C16	1.39601	C13′-C16′	1.39680
C11-C14	1.42227	C11′-C14′	1.42244
C14-C15	1.40460	C14′-C15′	1.40797
C15-C16	1.39382	C15′-C16′	1.39170
C14-N2	1.39592	C14′-N2′	1.40368
N2-C17	1.27960	N2′-C17′	1.27947
C17-C18	1.46174	C17′-C18′	1.45903
C18-S2	1.75088	C18′-S2′	1.75355
C18-C19	1.38645	C18′-C19′	1.38542
C19-C20	1.40827	C19′-C20′	1.40816
C20-C21	1.38631	C20′-C21′	1.38710
S2-C21	1.75885	S2′-C21′	1.75909
C21-C22	1.40339	C21′-C22′	1.40363
C22-C23	1.21935	C22′-C23′	1.21964
C23-C24	1.41957	C23′-C24′	1.42004
C24-C25	1.41173	C24′-C25′	1.41177
C24-C26	1.41240	C24′-C26′	1.41209
**ANGLE**	**o-PDT**	**ANGLE**	**o-PDT**
C6-S1-C9	91.64282	C6′-S1′-C9′	91.83895
C4-C5-C6	176.76564	C4′-C5′-C6′	170.61957
C9-C10-N1	133.41464	C9′-C10′-N1′	123.84971
C10-N1-C11	126.44810	C10′-N1′-C11′	117.15231
C14-N2-C17	126.71523	C14′-N2′-C17′	125.81983
N2-C17-C18	134.24868	N2′-C17′-C18′	132.84811
C18-S2-C21	91.68627	C18′-S2′-C21′	91.56327
C22-C23-C24	177.23143	C22′-C23′-C24′	173.33124
**BOND**	**m-PDT**	**BOND**	**m-PDT**
C1-C2	1.41037	C1′-C2′	1.40985
C2-C3	1.41164	C2′-C3′	1.41149
C2-C4	1.42061	C2′-C4′	1.42256
C4-C5	1.21877	C4′-C5′	1.21864
C5-C6	1.40402	C5′-C6′	1.40650
C6-C7	1.38601	C6′-C7′	1.38533
C6-S1	1.75682	C6′-S1′	1.75705
C7-C8	1.40863	C7′-C8′	1.41379
C8-C9	1.38577	C8′-C9′	1.38230
S1-C9	1.74922	S1′-C9′	1.74531
C9-C10	1.46010	C9′-C10′	1.44629
C10-N1	1.27938	C10′-N1′	1.28368
N1-C11	1.40631	N1′-C11′	1.40182
C11-C12	1.40780	C11′-C12′	1.40437
C12-C13	1.39011	C12′-C13′	1.39162
C13-C16	1.39457	C13′-C16′	1.39392
C11-C14	1.40066	C11′-C14′	1.40561
C14-C15	1.40539	C14′-C15′	1.40261
C15-C16	1.40518	C15′-C16′	1.40446
C15-N2	1.40487	C15′-N2′	1.40927
N2-C17	1.28431	N2′-C17′	1.28055
C17-C18	1.44484	C17′-C18′	1.45943
C18-S2	1.74615	C18′-S2′	1.74993
C18-C19	1.38320	C18′-C19′	1.38594
C19-C20	1.41030	C19′-C20′	1.40873
C20-C21	1.38751	C20′-C21′	1.38691
S2-C21	1.75824	S2′-C21′	1.75567
C21-C22	1.40311	C21′-C22′	1.40336
C22-C23	1.21938	C22′-C23′	1.21918
C23-C24	1.41902	C23′-C24′	1.41914
C24-C25	1.41191	C24′-C25′	1.41218
C24-C26	1.41202	C24′-C26′	1.41146
**ANGLE**	**m-PDT**	**ANGLE**	**m-PDT**
C6-S1-C9	91.58602	C6′-S1′-C9′	91.25115
C4-C5-C6	176.60894	C4′-C5′-C6′	174.54395
C9-C10-N1	133.49609	C9′-C10′-N1′	120.67546
C10-N1-C11	125.00617	C10′-N1′-C11′	121.79354
C15-N2-C17	119.65096	C15′-N2′-C17′	122.90429
N2-C17-C18	122.41205	N2′-C17′-C18′	132.54236
C18-S2-C21	91.21184	C18′-S2′-C21′	91.60045
C22-C23-C24	176.53157	C22′-C23′-C24′	176.52971

**Table 3:** Bond lengths for ***o*-ZnPDT** and ***m*-ZnPDT**

BOND	*o*-ZnPDT	BOND	*o*-ZnPDT
C1-C2	1.42186	C1′-C2′	1.42085
C2-C3	1.42200	C2′-C3′	1.42203
C2-C4	1.39809	C2′-C4′	1.40421
C4-C5	1.23096	C4′-C5′	1.22642
C5-C6	1.37774	C5′-C6′	1.38966
C6-C7	1.41401	C6′-C7′	1.39793
C6-S1	1.76622	C6′-S1′	1.76099
C7-C8	1.38077	C7′-C8′	1.39901
C8-C9	1.41980	C8′-C9′	1.39235
S1-C9	1.77302	S1′-C9′	1.74363
C9-C10	1.39719	C9′-C10′	1.45137
C10-N1	1.34876	C10′-N1′	1.28973
N1-C11	1.41129	N1′-C11′	1.38923
N1-Zn	1.94290	——	—–
C11-C12	1.40441	C11′-C12′	1.41240
C12-C13	1.38909	C12′-C13′	1.38661
C13-C16	1.39950	C13′-C16′	1.39841
C11-C14	1.42490	C11′-C14′	1.43019
C14-C15	1.40415	C14′-C15′	1.40692
C15-C16	1.38940	C15′-C16′	1.39140
C14-N2	1.42133	C14′-N2′	1.39140
N2-C17	1.32037	N2′-C17′	1.28116
N2-Zn	1.94290	——-	——-
Zn-O1	2.04892	——-	——-
Zn-O2	2.01692	——-	——-
C17-C18	1.40998	C17′-C18′	1.46033
C18-S2	1.76353	C18′-S2′	1.74750
C18-C19	1.39872	C18′-C19′	1.38738
C19-C20	1.39833	C19′-C20′	1.40658
C20-C21	1.39748	C20′-C21′	1.38911
S2-C21	1.77176	S2′-C21′	1.75841
C21-C22	1.38698	C21′-C22′	1.40041
C22-C23	1.22618	C22′-C23′	1.22148
C23-C24	1.40883	C23′-C24′	1.41390
C24-C25	1.41651	C24′-C25′	1.41519
C24-C26	1.41648	C24′-C26′	1.41707
**ANGLE**	***o*-ZnPDT**	**ANGLE**	***o*-ZnPDT**
C6-S1-C9	91.15801	C6′-S1′-C9′	91.21665
C4-C5-C6	169.26435	C4′-C5′-C6′	174.68969
C9-C10-N1	131.28692	C9′-C10′-N1′	131.44237
C10-N1-C11	123.47215	C10′-N1′-C11′	124.63288
N1-Zn-N2	89.22684	——-	——-
C11-N1-Zn	107.56032	——-	——-
C14-N2-Zn	107.03021	——-	——-
O1-Zn-O2	98.13725	——-	——-
C14-N2-C17	122.32526	C14′-N2′-C17′	126.49626
N2-C17-C18	122.32526	N2′-C17′-C18′	117.88437
C18-S2-C21	92.11041	C18′-S2′-C21′	91.43543
C22-C23-C24	170.30390	C22′-C23′-C24′	174.24680
**BOND**	***m*-ZnPDT**	**BOND**	***m*-ZnPDT**
C1-C2	1.41236	C1′-C2′	1.41337
C2-C3	1.41255	C2′-C3′	1.41224
C2-C4	1.42046	C2′-C4′	1.41906
C4-C5	1.22047	C4′-C5′	1.22193
C5-C6	1.39833	C5′-C6′	1.39501
C6-C7	1.39135	C6′-C7′	1.37504
C6-S1	1.76394	C6′-S1′	1.80502
C7-C8	1.40218	C7′-C8′	1.42303
C8-C9	1.39395	C8′-C9′	1.37409
S1-C9	1.75922	S1′-C9′	1.79440
C9-C10	1.41723	C9′-C10′	1.46857
C10-N1	1.30903	C10′-N1′	1.27878
N1-C11	1.44019	N1′-C11′	1.37785
C11-C12	1.39640	C11′-C12′	1.41038
C12-C13	1.40451	C12′-C13′	1.38970
C13-C16	1.39251	C13′-C16′	1.40912
C11-C14	1.40642	C11′-C14′	1.41427
C14-C15	1.40185	C14′-C15′	1.42512
C15-C16	1.41026	C15′-C16′	1.38499
C15-N2	1.38870	C15′-N2′	1.43247
N2-C17	1.27802	N2′-C17′	1.31627
C17-C18	1.48828	C17′-C18′	1.40972
C18-S2	1.78533	C18′-S2′	1.75903
C18-C19	1.37287	C18′-C19′	1.39911
C19-C20	1.42486	C19′-C20′	1.39690
C20-C21	1.37495	C20′-C21′	1.39669
S2-C21	1.78017	S2′-C21′	1.75919
C21-C22	1.40513	C21′-C22′	1.39778
C22-C23	1.22401	C22′-C23′	1.22084
C23-C24	1.42425	C23′-C24′	1.41973
C24-C25	1.41129	C24′-C25′	1.41229
C24-C26	1.41258	C24′-C26′	1.41232
N1-Zn1	1.98962	S1′-Zn2	2.40675
Zn1-O1	2.01367	Zn2-O3	2.00445
Zn1-O2	2.01418	Zn2-N2′	1.99121
**ANGLE**	***m*-ZnPDT**	**ANGLE**	***m*-ZnPDT**
C6-S1-C9	91.50628	C6′-S1′-C9′	92.36433
C4-C5-C6	178.39556	C4′-C5′-C6′	176.19523
C9-C10-N1	127.20977	C9′-C10′-N1′	133.11961
C10-N1-C11	121.37813	C10′-N1′-C11′	125.48684
C15-N2-C17	126.38251	C15′-N2′-C17′	122.13497
N2-C17-C18	133.58092	N2′-C17′-C18′	126.67908
C18-S2-C21	92.97866	C18′-S2′-C21′	91.15672
C22-C23-C24	174.32403	C22′-C23′-C24′	176.68674
C10-N1-Zn1	176.68674	S1′-Zn2-N2′	132.89784
C11-N1-Zn1	91.95766	S1′-Zn2-O3	110.41066
N1-Zn1-O1	121.11266	N2′-Zn2-O3	116.31018
N1-Zn1-O2	105.11015	——	—–

**Table 4:** Graphic comparison between the electronic excitations corresponding to the previously published molecule and the new synthesized molecule not analyzed.

**Table 5:** Review on Porphyrins used as photosensitized material. [2], [3], [4], [5], [6], [7], [8]

**Table 6:** Metal-free used as photosensitized material. [9], [10], [11], [12], [13], [14], [15]

(–): It is nor mentioned in the article

Mass spectrum m/z for the new molecule derivated of diethynylphenylthiophene

## 2 Experimental design and methods

The density functional theory (DFT) approximation as implemented in Gaussian 09, was used for all calculations that were carried out using the B3LYP functional and the 6-31g (2d,p) basis set. Full geometry optimization without symmetry constraints were carried out for all the stationary points. Harmonic frequency analysis allowed us to verify the optimized minima. The local minima were identified when the number of imaginary frequencies is equal to zero. Theoretically, the intensity of the band is expressed in terms of the oscillator strengths (f). Stationary points were modeled in the gas phase (vacuum). The analysis of the changes in electron density for a given electronic transition was based on the electron density difference maps (EDDMs) constructed using the GaussSum suite of programs. The Donor- aceptor capacity is relationshiped to TiO_2_, •OH, •OOH, and PD spectators. The photo-induced excitations of sunlight occur in the donor material. These excitons disseminate the scope of a donor / acceptor interface, where the transfer of electrons to the acceptor takes place. The Fig. 7 allows to reader understad the donate photogenerated electrons to diatomic oxygen to form the superoxide radical anion that can degrades the structure. The scheme of electronic traffic through a sensitized material is builded for understanding the electronic properties between Donor- Acceptor, which will allow stablish the capacity of the molecule in function of the HOMO- LUMO levels. Finally, the IR and Mass- spectrum were collected in the Spectrophometric Agilent Cary 630 FTIR with Attenuated Total Reflectance (ATR) and GC- MS Perkin Elmer Clarus 600 T- INTEC.

## Conflict of Interest

The authors declare that they have no known competing financial interests or personal relationships that could have appeared to influence the work reported in this paper.
